# Hepatic Artery Aneurysms as a Rare but Important Cause of Abdominal Pain; a Case Series

**Published:** 2019-04-06

**Authors:** Hamidreza Haghighatkhah, Morteza Sanei Taheri, Seyed Mohammadhadi Kharazi, Maryam Zamini, Sahar Rabani Khorasgani, Zahra Jahangiri Zarkani

**Affiliations:** 1 Radiology Department, Shohadaye Tajrish Hospital, Shahid Beheshti University of Medical Sciences, Tehran, Iran.

**Keywords:** Aneurysm, hepatic artery, abdominal pain, abdomen, acute, angiography

## Abstract

Hepatic artery aneurysm (HAA) is the common visceral aneurysms with the highest reported rate of rupture. The clinical manifestations depending on the size of the aneurysm include epigastric pain, obstruction of biliary tract, rupture and death. Imaging modalities like computed tomography (CT) scan and CT-angiography have a valuable role in the early detection of HHAs. Complications and selecting appropriate treatments depending on the size and location of the aneurysms. This article aimed to report clinical presentation, imaging finding and treatment of some patients presenting with HAAs to emergency department.

## Introduction:

Hepatic artery aneurysm (HAA) is a rare disease (0.002%–0.4%) but is a clinically important phenomenon (1). HAAs are traditionally the second most common visceral aneurysms with an incidence of 20% and have the highest (44%) reported rate of rupture (2). The clinical manifestations depending on the size of the aneurysm include epigastric pain, obstruction of biliary tract, rupture and death. Imaging modalities like computed tomography (CT) and CT-angiography have a valuable role in the early detection of HHA, its complications, and selecting appropriate treatments depending on the size and location of the aneurysms. In this case series, we reported 5 HAA cases with different etiologies, presentations and treatments.

## Case presentations:

***Case 1***

**Figure 1:**
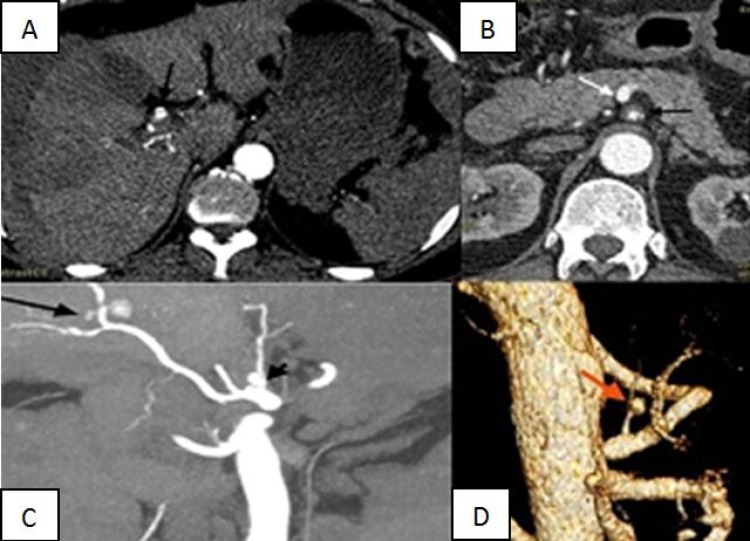
A) Axial contrast-enhanced CT (CECT) scan demonstrate contrast filled saccular out-pouching at the level of the left hepatic artery (black arrow). Hemoperitoneum with areas of higher attenuation indicates acute active bleeding is present. Peripheral segmental liver hypo-densities due to de-vascularization and vasospasm are also evident. B) Axial CECT through the celiac trunk level shows left gastric artery aneurysm with intraluminal thrombosis. C) Coronal Maximum Intensity Projection (MIP) reformation demonstrates concomitant aneurysm of hepatic artery (long arrow) and left gastric artery (short arrow). D) A volume-rendered 3D CT angiography image displays a small saccular left gastric artery aneurysm

The first case was a 50-year-old man with acute-onset epigastric and right upper abdominal pain. Abdominal ultrasonography demonstrated free abdominal fluid with internal clots. CT angiography was performed, which revealed a ruptured proper HAA with hemo-peritoneum in perihepatic space, para-colic gutters, and massive abdominopelvic hematoma. A simultaneous visceral aneurysm was also detected at the origin of the left gastric artery. The exploratory laparotomy revealed 2.5 liters of hemo-peritoneum, a ruptured aneurysmal sac in proximal of the left branch of proper hepatic artery with surrounding clots and intact liver parenchyma. Ligation in the proximal and distal parts of artery was done (Figure 1).

***Case 2***

**Figure 2:**
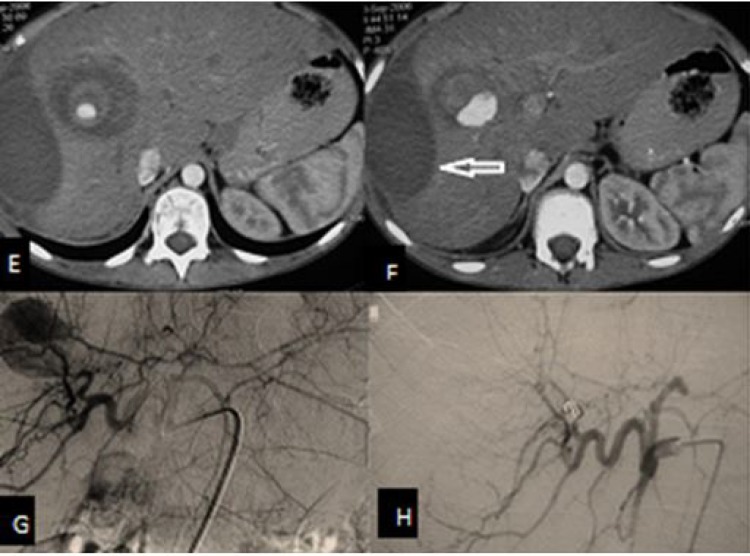
E, F) Axial contrast-enhanced CT (CECT) scan in arterial phase reveals out-pouching pseudo-aneurysm in hepatic artery branch along with intra parenchymal and sub-capsular liver hematoma (arrow). G) Selective digital subtraction angiography (DSA) from hepatic artery shows large pseudo-aneurysm in right hepatic artery. The patient underwent coil embolization for treatment of aneurysm. H) Aneurysm disappears after embolization

The second patient was an 11-year-old boy with history of falling and blunt trauma to the flank and formation of liver hematoma. Three weeks later, he was referred to emergency department with the chief complaint of abdominal pain. After proving pseudo-HAA in contrast-enhanced computed tomography (CECT) scan, the patient was referred for catheter base angiography and treated with coiled embolization (Figure 2).

***Case 3***

**Figure 3:**
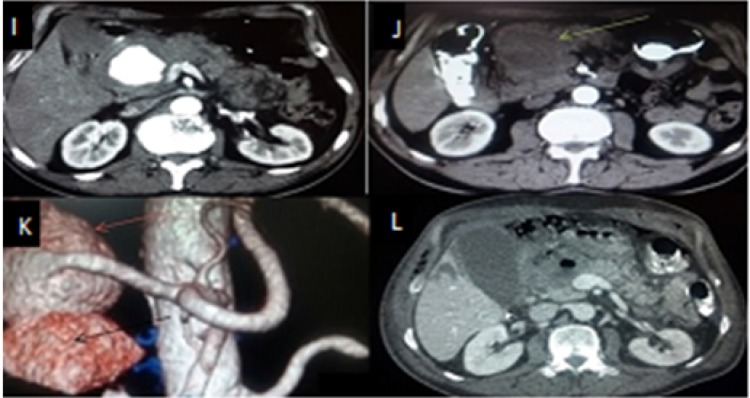
I, J) Axial computed tomography (CT) angiogram showing spontaneous saccular aneurysm in proper hepatic artery with peripheral thrombosis and internal diameter of 44 mm, and evidence of hematoma (white arrow) with compression to second part of duodenum and the head of pancreas. K) Volume-rendered 3D CT angiography image displays aneurysm from common hepatic artery (red arrow) and hematoma (black arrow) below it. L) The patient was referred one week after surgical repairing following abdominal pain. Contrast-enhanced CT (CECT) scan finding showed necrotizing center with severe edema around the head of pancreas with extension to porta-hepatis, peri-portal, and anterior sub-hepatic space suggestive of acute necrotizing pancreatitis

The patient was a 66-year-old male with epigastric pain and nausea for 10 days. Past medical history revealed smoking with hypertension. CT angiography showed saccular aneurysm in proper hepatic artery. The patient underwent surgical repair. A 70 × 70 mm aneurysmal lesion at the origin of proper hepatic artery was found in lesser sac and gastro-hepatic ligament. After attaining control of proper hepatic artery, end-to-end bypass graft was done in hepatic artery using saphenous vein. After 12^th^ post-operative day hospitalization, the patient referred to the hospital a week later with severe abdominal pain and vomiting. In CT scan, there was evidence of acute necrotizing pancreatitis, along with collection in liver. The patient was successfully managed with conservative treatment and discharged after two weeks without major complication (Figure 3).

***Case 4***

**Figure 4:**
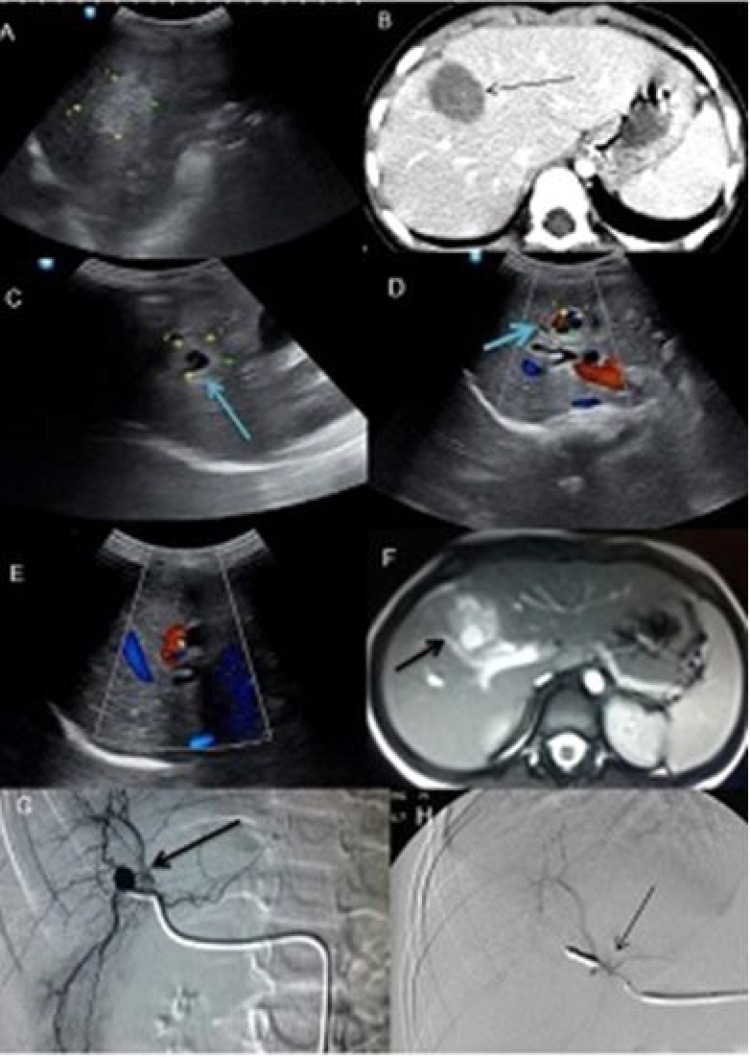
A) Ultrasound showed a hyper echo heterogeneous ill-defined lesion (arrow) in the right lobe of liver measuring 30*40 mm in favor of hematoma. B) Axial contrast-enhanced CT scan correlated with ultrasound finding (arrow). The patient underwent conservative treatment. C, D, E) One month later, ultrasound revealed a 23*23 mm hyper echo lesion with central 14*14 mm hypo echo vascular area in segment 8 of liver (an aneurysmal lesion). F) Axial T2W MRI showed a round hyper signal lesion (arrow), the same signal with aorta. G, H) Selective digital subtraction angiography (DSA) from right hepatic artery shows pseudo-aneurysm in right hepatic artery. The patient underwent coil embolization for treatment of aneurysm (arrow) and aneurysm disappeared after embolization (arrow)

The patient was a 4-year-old boy with history of blunt trauma to his right flank. One month later, the patient admitted with melena, hematemesis, and epigastric pain. Color Doppler ultrasonography, CECT scan, and magnetic resonance imaging (MRI) showed HAA. The patient was treated successfully with coiled embolization (Figure 4).

***Case 5***

The patient was a 24-year-old male with history of gunshot traumatization and surgery due to hepatic artery aneurysm and liver hematoma. He was referred due to re-bleeding and large liver-infected hematoma. The patient underwent successful coil embolization similar to case 2.

**Figure 5:**
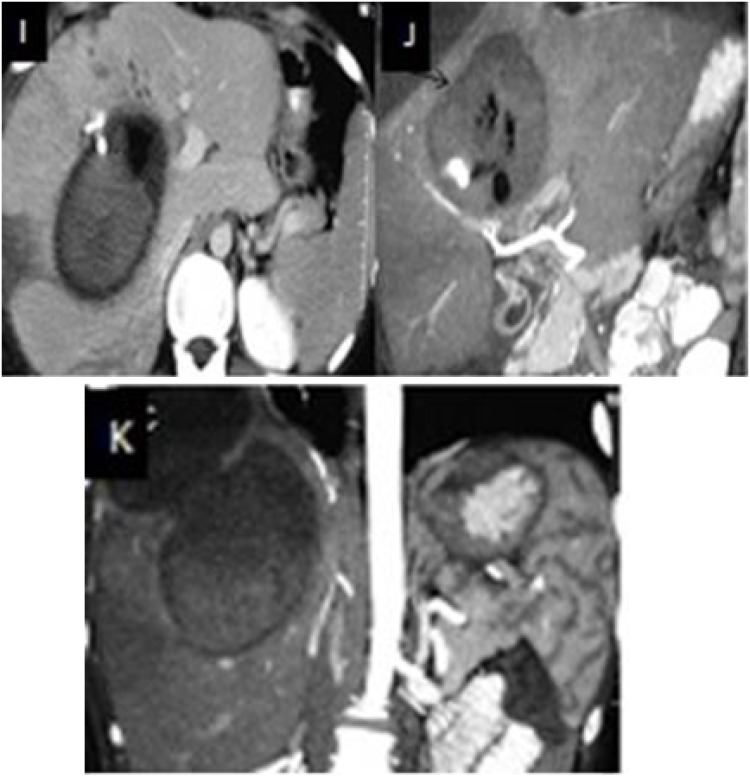
I, J, K) Axial contrast enhanced computed tomography (CECT) scan in arterial phase and coronal reformatted images from liver show pseudo-aneurysm in right hepatic artery (arrow) with infected liver hematoma containing gas density and also sub-capsular hematoma

## Discussion:

True HAAs are mostly due to degenerative or dysplastic change of the extrahepatic vessels. Their main cause is atherosclerosis (3), although vasculitis have also been reported (4). Pseudo-HAAs, accounting for approximately 20% of all HAAs (5), can be as intrahepatic or extrahepatic. Approximately 80% of aneurysms of the hepatic artery are extrahepatic which are mostly spontaneous, usually due to immunosuppression, biloma, and biliary tract infection (2, 6). The causes of intrahepatic pseudo-aneurysms are usually iatrogenic (liver transplantation, cholecystectomy, etc.), although they still have a potential for severe hemorrhage (6). 

The clinical manifestations tend to be non-specific, depending on the size of the aneurysm. Although small HAAs are often asymptomatic, the natural history is for progressive enlargement with increasing risk of rupture and death. HAAs may cause right upper quadrant and epigastric pain or obstruction of biliary tract. The classic triad of Quinke's, i.e., obstructive jaundice, abdominal pain and hemobilia, is seen in 30% of the patients (7). 

Inflammation associated with septic emboli may erode the arterial wall and then prompt HAA rupture, causing hemobilia (8). The presence of a common channel for both pancreatic and biliary ducts with no accessory duct drainage is predisposed to the development of acute pancreatitis due to the blockage of the pancreatic duct by blood clot (9). 

Multiple diagnosis tests are used, such as abdominal ultrasound, CT, CT angiography, MRI, endoscopy and angiography. Angiography is a therapeutic modality of choice in splanchnic aneurysms through embolization (10). The sensitivity and specificity of multi-detector computed tomographic angiography to diagnose aneurysm of the hepatic arteries was 100% (11).

The size and location of the aneurysm, patient age and comorbidities have critical role in determining the specific approach (12). Trans-arterial embolization (TAE) has a high rate of success for all causes of HAA (13). For intrahepatic aneurysms, embolization is the accepted treatment (3). Surgical treatment of ruptured HAAs can be allocated when the patient is in an unstable condition, the aneurysm is extrahepatic or larger than 2 cm, and endovascular intervention fails (1). Lee et al. reported an incidental large HAA and the patient was undergone open surgery for aneurysmectomy and the proper hepatic artery was anastomosed with gastrodeodenal artery (14). 

## Conclusion:

Visceral aneurysms such as hepatic artery aneurysm should be considered as a rare but important differential diagnosis of acute abdominal pain. All emergency physicians and surgeons should kept this diagnosis n their mind to can prevent from its life threatening complications. 
